# Are MRI high-signal changes of alar and transverse ligaments in acute whiplash injury related to outcome?

**DOI:** 10.1186/1471-2474-11-260

**Published:** 2010-11-11

**Authors:** Nils Vetti, Jostein Kråkenes, Geir E Eide, Jarle Rørvik, Nils E Gilhus, Ansgar Espeland

**Affiliations:** 1 Department of Radiology, Haukeland University Hospital, Bergen, Norway; 2 Section for Radiology, Department of Surgical Sciences, University of Bergen, Bergen, Norway; 3 Centre for Clinical Research, Haukeland University Hospital, Bergen, Norway; 4 Department of Public Health and Primary Health Care, University of Bergen, Norway; 5 Department of Neurology, Haukeland University Hospital, Bergen, Norway; 6 Department of Clinical Medicine, University of Bergen, Bergen, Norway

## Abstract

**Background:**

Upper neck ligament high-signal changes on magnetic resonance imaging (MRI) have been found in patients with whiplash-associated disorders (WAD) but also in non-injured controls. The clinical relevance of such changes is controversial. Their prognostic role has never been evaluated. The purpose of this study was to examine if alar and transverse ligament high-signal changes on MRI immediately following the car accident are related to outcome after 12 months for patients with acute WAD grades 1-2.

**Methods:**

Within 13 days after a car accident, 114 consecutive acute WAD1-2 patients without prior neck injury or prior neck problems underwent upper neck high-resolution proton-weighted MRI. High-signal changes of the alar and transverse ligaments were graded 0-3. A questionnaire including the impact of event scale for measuring posttraumatic stress response and questions on patients' expectations of recovery provided clinical data at injury. At 12 months follow-up, 111 (97.4%) patients completed the Neck Disability Index (NDI) and an 11-point numeric rating scale (NRS-11) on last week neck pain intensity. Factors potentially related to these outcomes were assessed using multiple logistic regression analyses.

**Results:**

Among the 111 responders (median age 29.8 years; 63 women), 38 (34.2%) had grades 2-3 alar ligament changes and 25 (22.5%) had grades 2-3 transverse ligament changes at injury. At 12 months follow-up, 49 (44.1%) reported disability (NDI > 8) and 23 (20.7%) neck pain (NRS-11 > 4). Grades 2-3 ligament changes in the acute phase were not related to disability or neck pain at 12 months. More severe posttraumatic stress response increased the odds for disability (odds ratio 1.46 per 10 points on the impact of event scale, p = 0.007) and so did low expectations of recovery (odds ratio 4.66, p = 0.005).

**Conclusions:**

High-signal changes of the alar and transverse ligaments close after injury did not affect outcome for acute WAD1-2 patients without previous neck problems. High-resolution upper neck MRI has limited value for the initial examination and follow-up of such patients.

## Background

The alar and transverse ligaments are important stabilizers at the craniovertebral junction [1-5] and can be injured during neck trauma [6-10]. These ligaments can be visualised on magnetic resonance imaging (MRI) [11-16]. High-signal changes of the alar and transverse ligaments on high-resolution MRI have been reported to be more frequent in chronic whiplash-associated disorders (WAD) compared to non-injured controls [17]. In the same study sample, such ligament changes were related to neck disability and trauma factors like impact direction and head position at the instant of collision [17-19]. These results have not been confirmed by others, and high-signal changes of upper neck ligaments are reported to be frequent also in asymptomatic and symptomatic non-injured controls [14,20-22]. Such changes thus have unclear cause and clinical relevance. They might be traumatic in some cases but might also represent pre-traumatic morphologic variants with loose connective tissue or fat interspersed between fibres [21-23]. If such variants affect ligament strength and prognosis after neck trauma these MRI findings could represent a target for interventions to improve patients' recovery.

The prognostic factors for developing chronic WAD after whiplash injury have not been established. Female gender, more severe post traumatic stress response, and reduced expectations of recovery have been found associated with poor outcome in WAD [24-28]. In recent reviews, high score of initial pain has been pointed out as the most important predictor for delayed recovery [29,30]. In prior MRI studies on WAD, traumatic findings in the acute phase of whiplash injury were rare [31-35] and did not affect recovery [31,32,35,36]. However, due to the magnetic field strength and MRI protocols chosen, the alar and transverse ligaments could not be assessed. Data on the prognostic role of MRI high-signal changes of these ligaments in acute WAD have been requested [32].

This prospective follow-up study included patients with acute WAD grade 1 or 2 as defined by the Quebec Task Force [37], that is acute neck complaints after whiplash trauma but no fractures, dislocations or neurological signs. All patients were examined with a dedicated high-resolution upper neck MRI protocol. The aim was to evaluate if high-signal changes of the alar and transverse ligaments in the acute phase of whiplash injury are related to outcome after 12 months.

## Methods

### Patients

From May 2007 until March 2009 114 acute WAD1-2 patients were recruited consecutively from a primary ward (Bergen Accident and Emergency Department) (n = 76) and a hospital clinic (Haukeland University Hospital) (n = 38). All patients underwent MRI of their upper neck ligaments. MRI findings in relation to clinical characteristics in the acute phase of injury of this inception cohort are reported elsewhere [38].

To be included, patients should be Norwegian-speaking drivers or passengers, aged 18-80 years, sustaining a car accident during the last 7 days, reporting onset of neck pain within 48 hours after the accident, and without any neurological signs or clinical or radiological signs of neck fracture or dislocation. First author ascertained the WAD grading by interviewing the patients and reviewing reports from clinicians and radiologists. The exclusion criteria were prior neck injury or whiplash trauma, prior neck problems (i.e. prior neck pain of more than 30 days in total or reported treatment for neck problems during the last 10 years), prior severe head injury, previous cervical spine surgery, rheumatic disease, cancer or any other serious somatic or psychiatric conditions, and pregnancy.

All participants were asked to complete a follow-up questionnaire 12 months after the accident. Three did not respond despite reminders and were excluded from the study, 111 (97.4%) responded and form the current study sample.

The Regional Committee for Medical Research Ethics, Western Norway Health Region approved this study. Written informed consent was obtained from all study participants.

### Clinical data - acute phase

Within 0 - 13 (median 4) days after their accident all patients filled in a questionnaire containing items regarding potential risk factors for developing chronic disability or pain in acute WAD1-2. It included an 11-point numeric rating scale (NRS-11) of average neck pain since injury (initial neck pain); 0 = no pain and 10 = worst possible pain [39,40], a pain drawing for the localization of maximum neck pain [41], and questions regarding accident-related factors and education. Patients' subjective reports of concomitant head injury were registered. Post traumatic stress response was evaluated by the impact of event scale (IES, theoretic range 0-75) [42], which has been validated in WAD [26,28,43]. The result was dichotomized into IES ≥ 26 and IES < 26 [28,43]. The mean value of completed questions replaced any missing items when calculating the total IES score. Patients also answered to what extent (little, some, great) they expected to get rid of their pain after the accident. These expectations of recovery were dichotomized into high (great extent) and low (little/some extent).

### MRI protocol

MRI was performed within 0 - 13 (median 5) days after the car accident (within 7 days in 96 patients, 86.5%) in a 1.5 Tesla scanner (Symphony Mastroclass, Siemens Medical System, Erlangen, Germany), using a standard one-channel receive-only head coil. Patients' head and neck were in a neutral position. To visualise the alar and transverse ligaments with high spatial resolution while maintaining adequate imaging contrast and signal to noise ratio, a pre-existing MRI protocol was used [23,44]. It included proton-density-weighted fast spin echo (FSE) sequences in three orthogonal planes, axial, coronal and sagittal: repetition time (TR)/echo time (TE) 2150-2660/15 ms, slice thickness 1.5 mm, interslice gap 0.0 mm or 0.3 mm (sagittal), field of view (FOV) 175 mm × 200 mm or 200 mm × 200 mm (coronal), voxel size 0.6-0.7 × 0.4 × 1.5 mm^3 ^and echo train length (ETL) 13. Two sagittal STIR sequences followed but these were not used in the present study. The summarized acquisition time for the 5 sequences was 31 min 5 s.

### MRI evaluation

The alar and transverse ligaments were graded 0-3 on the proton sequences based on the ratio between any high-signal part and the total cross-sectional area of the ligament as judged visually [17,23,45]. No high signal was graded 0, high signal in 1/3 or less of the total cross section was graded 1, high signal in 1/3 to 2/3 of the total cross section was graded 2, and high signal in 2/3 or more of the total cross section was graded 3. The right and left sides were graded separately. The image with the largest cross-sectional area of high signal was used for grading, alar ligaments on sagittal sections and transverse ligaments on sagittal or coronal sections. Any high signal had to be seen in at least two imaging planes to be graded 1-3; otherwise it was graded 0 (no high signal). Homogenous grey ligaments were graded 2.

Two radiologists (6 and 26 years experience) who were blinded to clinical data independently graded all proton images, which were de-identified and presented in a random order interspersed between images of non-injured individuals. Both radiologists thereafter solved all disagreements by consensus reading of images. Their consensus grading was used in the analysis, where grades 2 and 3 were combined into one category. Disagreement on the presence of grades 2-3 changes per patient concerned 13 (11.7%) patients for the alar ligaments and 19 (17.1%) patients for the transverse ligament. Kappa for interobserver agreement on presence of grades 2-3 changes was 0.73 for the alar ligaments and 0.52 for the transverse ligament.

### Clinical outcome data

Uninformed of their MRI results, patients filled in the follow-up questionnaire 51-56 (median 52) weeks after the accident. Primary outcome was neck disability as measured by a modified version of the Neck Disability Index (NDI) [46-48]. NDI should be calculated only when at least 8 of 10 items are answered and was given as a percentage of the highest achievable score [46]. According to previously validated cut off values, NDI was dichotomized into NDI ≤ 8% or NDI > 8% [48-50]. Neck pain during the preceding week was registered on an NRS-11 and categorized into NRS-11 0-4 or NRS-11 5-10 [32,39]. All 111 patients returned valid data for both NDI and neck pain.

### Statistical analyses

Fisher's exact test was used to compare proportions between groups. To compare means the Mann-Whitney U test was used as normality could not be assumed. Multiple logistic regression analyses (stepwise backward, using likelihood-ratio tests) were performed with respectively NDI and neck pain NRS-11 as binary outcome variables. In these regression analyses mutual adjustments were done for age and gender and for all factors potentially related to outcome with p < 0.2 in the crude analysis. Interaction between variables significantly related to outcome was looked for. SPSS 16.0 was used to analyze data. *P *≤ 0.05 indicated statistical significance.

According to sample size calculations (significance level 5%, power 80%), assuming that one third of the acute WAD1-2 patients would show ligament high-signal changes, a total of 100 responders at 12 months follow-up would be needed to detect a difference in proportions recovered from 60% in those without ligament changes to 30% in those with ligament changes as statistically significant.

## Results

### Patient characteristics - acute phase

**Figure 1:**
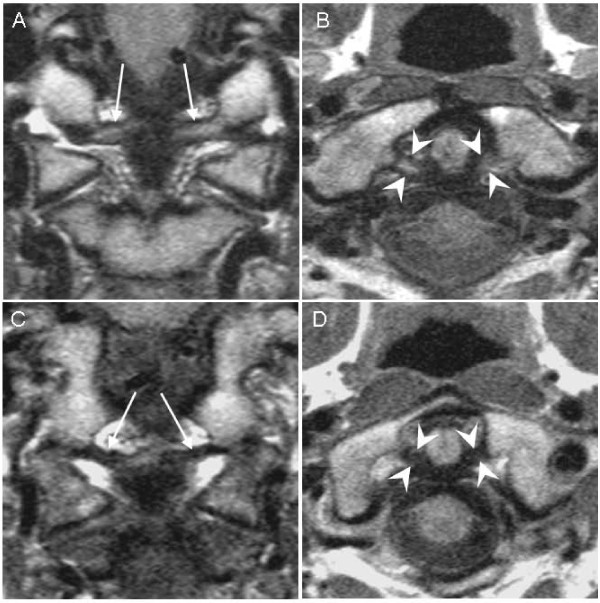
**MRI of alar and transverse ligaments**. High-resolution proton-weighted MRI sections of upper neck alar and transverse ligaments. Grade 3 alar ligament high-signal changes (arrows) on coronal section (A) and grade 2 transverse ligament high-signal changes (arrowheads) on axial section (B) in two patients recovered (NDI ≤ 8%) at follow-up. Grade 0 alar ligaments (arrows) on coronal section (C) and grade 0 transverse ligament (arrowheads) on axial section (D) in two different patients reporting disability (NDI > 8%) at follow-up for comparison.

Median age of the 111 patients was 29.8 years, and 63 patients (56.8%) were women (Table 1). Fifty patients (45.0%) had initial neck pain NRS-11 > 4, and 36 patients (32.4%) had IES score ≥ 26. MRI in the acute phase of injury showed grades 2-3 alar ligament changes in 38 (34.2%) of the 111 patients and grades 2-3 transverse ligament changes in 25 (22.5%) (Figure 1).

**Table 1:** Clinical data and MRI ligament findings at injury of 111 WAD1-2 patients

	N	%	Median (range)
Clinical characteristics			
Women	63	56.8	
Age, years			29.8 (18.1-69.2)
Higher education (> 12 years)	50	45.0	
Initial neck pain intensity, NRS-11 score (0 to 10)			4.0 (1.0 - 9.0)
Time accident - onset neck pain, hours			0.5 (0.0-48.0)
Pain maximum in upper neck (n = 105)	41	39.0	
Post traumatic stress, IES score (0 to 75)			19.0 (0.0-67.0)
High expectation of recovery (vs. low)	90	81.1	
Time accident - MRI, days			5.0 (0.0-13.0)
Accident-related factors			
Impact direction			
Rear-end collision	69	62.2	
Front-end collision	25	22.5	
Side impact collision	10	9.0	
Other (e.g. roll-over, complex)	7	6.3	
Head turned at impact (n = 93)	29	31.2	
Head injury at accident	13	11.7	
Seat belt used at impact	105	94.6	
Head restraint present at impact (n = 107)	94	87.9	
Airbag deployment at impact (n = 110)	15	13.6	
Patient car speed at impact, km/h (n = 109)			0.0 (0.0 - 75.0)
Relative car speed* at impact, km/h (n = 84)			45.0 (10.0-150.0)
MRI ligament findings			
Grades 2-3 alar ligament changes†	38	34.2	
Grades 2-3 transverse ligament changes†	25	22.5	

MRI = magnetic resonance imaging; WAD = whiplash-associated disorders; NRS-11 = 11-point numeric rating scale.

*Difference between vehicle speeds if rear end collision, otherwise sum of vehicle speeds.

† Highest assigned grade if different between right and left side.

### Unadjusted outcome analyses

At 12 months follow-up, 49 (44.1%) patients had NDI > 8% and 23 (20.7%) had neck pain NRS-11 > 4. In unadjusted analyses (Table 2), these outcomes were not significantly related to MRI grades 2-3 changes of alar (p = 0.14-0.23) or transverse ligaments (p = 0.49-0.59) in the acute phase.

**Table 2:** Disability and pain outcomes for 111 WAD1-2 patients at 12 months follow-up

	NDI score > 8%		NRS-11 neck pain score > 4	
	
	%	p*	%	p*
Gender		0.125		0.032
Women	50.8		28.6	
Men	35.4		10.4	
Age, years		0.286		0.759
< 20	50.0		14.3	
20-30	38.6		22.7	
30-40	34.8		13.0	
40-50	63.6		27.3	
>50	37.5		25.0	
Initial pain		0.034		<0.001
NRS-11 score ≤ 4	34.4		8.2	
NRS-11 score > 4	56.0		36.0	
Post traumatic stress		0.015		0.011
IES score < 26	36.0		13.3	
IES score ≥ 26	61.1		36.1	
Expectations of recovery		0.001		0.013
High	36.7		15.6	
Low	76.2		42.9	
Grades 2-3 MRI alar ligament changes†		0.229		0.143
No	39.7		16.4	
Yes	52.6		28.9	
Grades 2-3 MRI transverse ligament changes†		0.493		0.588
No	41.9		22.1	
Yes	52.0		16.0	

WAD = whiplash-associated disorders; NDI = neck disability index; NRS-11 = 11-point numeric rating scale; IES = impact of event scale; MRI = magnetic resonance imaging.

* P values are based the difference in outcome proportions between the categories as calculated by the Fisher's exact test.

† Highest assigned grade if different between right and left side.

The risk of disability (NDI > 8%) increased with initial neck pain NRS-11 > 4 (p = 0.034), post traumatic stress response IES score ≥ 26 (p = 0.015), and low expectations of recovery (p = 0.001). Also when treating continuous explanatory variables uncategorized, the risk of disability increased with initial neck pain NRS-11 scores (p = 0.011) and IES scores (p = 0.002). Risk factors for neck pain (NRS-11 > 4) were the same as for disability but in addition included female gender (p = 0.032) (Table 2). No other clinical or accident-related characteristic given in table 1 was related to disability or neck pain at follow-up with p < 0.2.

### Adjusted outcome analyses

In the adjusted logistic regression analysis (Table 3) higher IES scores (odds ratio (OR) per 10 IES points 1.46) and low expectations of recovery (OR 4.66) in the acute phase of injury were related to NDI > 8% at 12 months. No interaction between these two explanatory variables was found. Female gender (OR 3.25), higher IES scores (OR per 10 IES points 1.93), and low expectations of recovery (OR 21.56) were related to neck pain NRS-11 > 4 (Table 3). In this model an interaction between expectations of recovery and posttraumatic stress was found. Post traumatic stress increased the risk of neck pain NRS-11 > 4 for patients with high expectations of recovery (OR 1.93 per 10 IES points) but not for patients with low expectations (OR 1.93 × 0.51 = 0.98 per 10 IES points).

**Table 3:** Logistic regression analysis using NDI and neck pain as 12 months outcome for 111 acute WAD1-2 patients

Explanatory variables	NDI score > 8%					NRS-11 neck pain score > 4				
	
	Unadjusted		Adjusted			Unadjusted		Adjusted		
	
	OR	p*	OR	95% CI	p*	OR	p*	OR	95% CI	p*
Gender, females vs. males	1.88	0.105			0.210†	3.44	0.016	3.25	(1.00,10.50)	0.038
Age, years	1.02	0.343			0.093†	1.02	0.310			0.070†
Initial pain, NRS-11 score	1.27	0.016			0.699†	1.50	0.001			0.267†
Posttraumatic stress, per 10 IES points	1.53	0.001	1.46	(1.10,1.94)	0.007	1.65	0.001	1.93	(1.24,3.00)	0.001
Expectation of recovery, low vs. high	5.53	0.001	4.66	(1.50,14.47)	0.005	4.07	0.009	21.56	(2.52,184.16)	0.006
Expectation of recovery × posttraumatic stress						1.23	0.160	0.51	(0.26,1.00)	0.048
Grades 2-3 alar ligament changes on MRI‡, (yes vs. no)						2.07	0.123			0.369†

NDI = neck disability index; WAD = whiplash-associated disorders; NRS-11 = 11-point numeric rating scale; OR = odds ratio; CI = confidence interval; IES = impact of event scale; MRI = magnetic resonance imaging.

* P values are based on likelihood-ratio test.

† Not in the final model, p-value for adding term to final model.

‡ Highest assigned grade if different between right and left side.

When included into these logistic regression models, MRI grades 2-3 ligament changes in the acute phase of injury were not related to NDI > 8% (alar: p = 0.76, transverse: p = 0.76) or neck pain NRS-11 > 4 (alar: p = 0.51, Table 3; transverse: p = 0.42) at follow-up.

## Discussion

In this first study on the prognostic value of upper neck ligament MRI findings, high-signal changes of the alar and transverse ligaments at injury were not related to outcome 12 months after whiplash injury. This result was highly robust and remained after adjustments for factors that may influence outcome. We hardly missed relevant high-signal changes, since every patient underwent dedicated MRI within 13 days (86.5% within 7 days) after the accident. The ligament grading had adequate reliability, was performed blinded to outcomes, and was not conveyed to the patients or their health care providers, since information *per se *on MRI results can affect prognosis [51].

The finding that ligament high-signal changes in acute WAD1-2 were not related to outcome has important implications. First, due to this lack of prognostic value, such changes are unlikely to represent a target for treatment, regardless of whether they are traumatic or represent morphologic ligament variants. Second, routine use of high-resolution upper neck MRI is not warranted in acute WAD1-2. Third, the high-signal changes are less likely to be injury-induced. Although structural damage from an injury may heal without causing long-term complaints, at least some prognostic effect of alar and transverse ligament high-signal changes would be expected if they were due to the acute, mechanic incident. The ligament changes more likely reflect normal variants, also because they were not related to trauma factors and were equally frequent in non-injured controls, as reported elsewhere [38]. Imaging artefacts or age dependent degeneration can not explain such high-signal changes [23]. Further data on the underlying morphology could provide insight into MRI evaluation of ligaments, but are unlikely to aid clinical decisions in acute WAD1-2.

The present study showed that female gender, more severe post traumatic stress response, and reduced expectations of recovery are associated with poor outcome in WAD, in line with previous reports [24-28]. An independent effect of degree of initial pain [24,29,30,43] was not confirmed, probably because pain just after the accident may be intense but temporary. Impact direction, head turned at impact or speed at impact did not affect outcomes, similar to previous findings on collision factors [24,29,30].

In this prospective study of unselected WAD1-2 patients without previous neck problems we found better outcomes than in two previous studies; one Australian study [43] reporting NDI > 8% in 60% at 24 months and one Danish study [32] reporting NRS-11 score > 4 in 44% at 12 months. This may be explained by their inclusion of patients at higher risk due to neurological signs [43] (WAD3) or more severe initial symptoms [32]. Patients with previous neck problems probably have poorer prognosis [29,52,53], and the prognosis after isolated whiplash trauma would best be ascertained in cohorts excluding such patients. However, due to recall bias we can not be sure that all our included WAD1-2 patients actually had no prior neck problems.

A major strength of our study is the prospective design and the high proportion of responders at follow-up (97%, 111/114) which prevented selection bias. Our sample of patients both from a primary ward and a hospital clinic should be representative of WAD1-2 patients reporting no previous neck problems who seek medical care shortly after a car accident. The numbers included should be adequate to detect clinical relevant differences in proportions recovered between patients with and without ligament high-signal changes. We had insufficient data to discriminate between WAD1 and WAD2. However, the effect of WAD grades on outcome is controversial [53-55]. Neither did we include data on anxiety, depression or cervical range of movement [43,56]. We had to limit and prioritize between possible risk factors according to sample size and distribution of outcome variables. Potential residual confounders could not have changed our results for prognostic value of MRI high-signal ligament changes unless they were unequally distributed between patients with and without such changes.

In contrast to previous examinations of acute WAD1-2 patients, our MRI protocol was intended to visualise craniovertebral ligaments. We focused only on the alar and transverse ligaments. Investigating the mid or lower neck ligaments or other anatomical structures at the cervical spine was beyond the scope of this study. Previous studies have focused on fracture or dislocation, traumatic disc or endplate changes, soft tissue bleeding/edema, posterior or anterior longitudinal ligament rupture and spinal cord injuries [31-36]. As no relation to prognosis was found, cervical spine MRI has not been recommended as a standard procedure in these patients [31-33]. Our results show that adding MRI sequences capable of visualising craniovertebral ligaments does not change these recommendations in acute WAD1-2.

Acute ligament injuries not visible on our MRI sequences might through a subsequent repair process of fibrosis and scarring cause high-signal changes at a later stage of whiplash injury. Results from case-controlled studies on ligament changes in chronic WAD are contradictory [17,21,22]. No relation between ligament changes and time since injury (40 days to 59 years, median 5 years) was found in clinically referred WAD1-2 patients [23]. However, acute WAD cohorts should be studied with MRI follow-up examinations to find out how ligament structure may alter over time. Such studies could help establishing if high-resolution upper neck MRI adds valuable information at a later stage of whiplash injury.

## Conclusions

In this study of acute WAD1-2 patients without previous neck problems, MRI high-signal changes of the alar and transverse ligaments in the acute phase were not related to disability or neck pain 12 months after injury. Female gender, more severe post traumatic stress response, and low expectations of recovery were associated with poor outcome at 12 months. Upper neck MRI is of limited value in the initial examination and follow-up of WAD1-2 patients, and is not recommended for routine use.

## Competing interests

The authors declare that they have no competing interests.

## Authors' contributions

All authors collaborated in designing the study. NV and JK performed the image interpretation. NV collected and coded all data. GEE, NV and AE performed the statistical analyses. All authors contributed in writing the manuscript and read and approved the final version.

## Pre-publication history

The pre-publication history for this paper can be accessed here:

http://www.biomedcentral.com/1471-2474/11/260/prepub

## References

[B1] Dvorak J Panjabi MM Functional anatomy of the alar ligaments Spine (Phila Pa 1976) 1987 12 183 189 3589810 10.1097/00007632-198703000-00016

[B2] Dvorak J Schneider E Saldinger P Rahn B Biomechanics of the craniocervical region: the alar and transverse ligaments J Orthop Res 1988 6 452 461 10.1002/jor.1100060317 3357093

[B3] Heller JG Amrani J Hutton WC Transverse ligament failure: a biomechanical study J Spinal Disord 1993 6 162 165 8504229

[B4] Panjabi M Dvorak J Crisco J III Oda T Hilibrand A Grob D Flexion, extension, and lateral bending of the upper cervical spine in response to alar ligament transections J Spinal Disord 1991 4 157 167 10.1097/00002517-199106000-00005 1806080

[B5] Saldinger P Dvorak J Rahn BA Perren SM Histology of the alar and transverse ligaments Spine (Phila Pa 1976) 1990 15 257 261 1693791 10.1097/00007632-199004000-00001

[B6] Adams VI Neck injuries: III. Ligamentous injuries of the craniocervical articulation without occipito-atlantal or atlanto-axial facet dislocation. A pathologic study of 21 traffic fatalities J Forensic Sci 1993 38 1097 1104 8228882

[B7] Dickman CA Greene KA Sonntag VK Injuries involving the transverse atlantal ligament: classification and treatment guidelines based upon experience with 39 injuries Neurosurgery 1996 38 44 50 10.1097/00006123-199601000-00012 8747950

[B8] Fielding JW Cochran GB Lawsing JF III Hohl M Tears of the transverse ligament of the atlas. A clinical and biomechanical study J Bone Joint Surg Am 1974 56 1683 1691 4434037

[B9] Obenauer S Herold T Fischer U Fadjasch G Koebke J Grabbe E Saternus KS The evaluation of experimentally induced injuries to the upper cervical spine with a digital x-ray technic, computed tomography and magnetic resonance tomography Rofo 1999 171 473 479 10668513 10.1055/s-1999-271

[B10] Saternus KS Thrun C Traumatology of the alar ligaments Aktuelle Traumatol 1987 17 214 218 2891250

[B11] Dickman CA Mamourian A Sonntag VK Drayer BP Magnetic resonance imaging of the transverse atlantal ligament for the evaluation of atlantoaxial instability J Neurosurg 1991 75 221 227 10.3171/jns.1991.75.2.0221 2072158

[B12] Kim HJ Jun BY Kim WH Cho YK Lim MK Suh CH MR imaging of the alar ligament: morphologic changes during axial rotation of the head in asymptomatic young adults Skeletal Radiol 2002 31 637 642 10.1007/s00256-002-0572-2 12395275

[B13] Pfirrmann CW Binkert CA Zanetti M Boos N Hodler J MR morphology of alar ligaments and occipitoatlantoaxial joints: study in 50 asymptomatic subjects Radiology 2001 218 133 137 11152791 10.1148/radiology.218.1.r01ja36133

[B14] Roy S Hol PK Laerum LT Tillung T Pitfalls of magnetic resonance imaging of alar ligament Neuroradiology 2004 46 392 398 10.1007/s00234-004-1193-3 15112112

[B15] Willauschus WG Kladny B Beyer WF Gluckert K Arnold H Scheithauer R Lesions of the alar ligaments. In vivo and in vitro studies with magnetic resonance imaging Spine (Phila Pa 1976) 1995 20 2493 2498 8610243 10.1097/00007632-199512000-00006

[B16] Wilmink JT Patijn J MR imaging of alar ligament in whiplash-associated disorders: an observer study Neuroradiology 2001 43 859 863 10.1007/s002340100600 11688704

[B17] Krakenes J Kaale BR Magnetic resonance imaging assessment of craniovertebral ligaments and membranes after whiplash trauma Spine (Phila Pa 1976) 2006 31 2820 2826 17108836 10.1097/01.brs.0000245871.15696.1f

[B18] Kaale BR Krakenes J Albrektsen G Wester K Whiplash-associated disorders impairment rating: neck disability index score according to severity of MRI findings of ligaments and membranes in the upper cervical spine J Neurotrauma 2005 22 466 475 10.1089/neu.2005.22.466 15853463

[B19] Kaale BR Krakenes J Albrektsen G Wester K Head position and impact direction in whiplash injuries: associations with MRI-verified lesions of ligaments and membranes in the upper cervical spine J Neurotrauma 2005 22 1294 1302 10.1089/neu.2005.22.1294 16305317

[B20] Baumert B Wortler K Steffinger D Schmidt GP Reiser MF Baur-Melnyk A Assessment of the internal craniocervical ligaments with a new magnetic resonance imaging sequence: three-dimensional turbo spin echo with variable flip-angle distribution (SPACE) Magn Reson Imaging 2009 27 954 960 10.1016/j.mri.2009.01.012 19282121

[B21] Dullerud R Gjertsen O Server A Magnetic resonance imaging of ligaments and membranes in the craniocervical junction in whiplash-associated injury and in healthy control subjects Acta Radiol 2010 51 207 212 10.3109/02841850903321617 19912072

[B22] Myran R Kvistad KA Nygaard OP Andresen H Folvik M Zwart JA Magnetic resonance imaging assessment of the alar ligaments in whiplash injuries: a case-control study Spine (Phila Pa 1976) 2008 33 2012 2016 18708935 10.1097/BRS.0b013e31817bb0bd

[B23] Vetti N Krakenes J Eide GE Rorvik J Gilhus NE Espeland A MRI of the alar and transverse ligaments in whiplash-associated disorders (WAD) grades 1-2: high-signal changes by age, gender, event and time since trauma Neuroradiology 2009 51 227 235 10.1007/s00234-008-0482-7 19083212

[B24] Berglund A Bodin L Jensen I Wiklund A Alfredsson L The influence of prognostic factors on neck pain intensity, disability, anxiety and depression over a 2-year period in subjects with acute whiplash injury Pain 2006 125 244 256 10.1016/j.pain.2006.05.026 16806708

[B25] Carroll LJ Holm LW Ferrari R Ozegovic D Cassidy JD Recovery in whiplash-associated disorders: do you get what you expect? J Rheumatol 2009 36 1063 1070 10.3899/jrheum.080680 19228657

[B26] Drottning M Staff PH Levin L Malt UF Acute Emotional Response to Common Whiplash Predicts Subsequent Pain Complaints - A Prospective-Study of 107 Subjects Sustaining Whiplash Injury Nord J Psychiatry 1995 49 293 299 10.3109/08039489509011919

[B27] Holm LW Carroll LJ Cassidy JD Skillgate E Ahlbom A Expectations for recovery important in the prognosis of whiplash injuries PLoS Med 2008 5 e105 10.1371/journal.pmed.0050105 18479182 PMC2375948

[B28] Kongsted A Bendix T Qerama E Kasch H Bach FW Korsholm L Jensen TS Acute stress response and recovery after whiplash injuries. A one-year prospective study Eur J Pain 2008 12 455 463 10.1016/j.ejpain.2007.07.008 17900949

[B29] Carroll LJ Holm LW Hogg-Johnson S Cote P Cassidy JD Haldeman S Nordin M Hurwitz EL Carragee EJ van d V Peloso PM Guzman J Course and prognostic factors for neck pain in whiplash-associated disorders (WAD): results of the Bone and Joint Decade 2000-2010 Task Force on Neck Pain and Its Associated Disorders Spine (Phila Pa 1976) 2008 33 Suppl 4 83 92 10.1097/BRS.0b013e3181643eb8 18204405

[B30] Scholten-Peeters GG Verhagen AP Bekkering GE van der Windt DA Barnsley L Oostendorp RA Hendriks EJ Prognostic factors of whiplash-associated disorders: a systematic review of prospective cohort studies Pain 2003 104 303 322 10.1016/S0304-3959(03)00050-2 12855341

[B31] Borchgrevink G Smevik O Haave I Haraldseth O Nordby A Lereim I MRI of cerebrum and cervical columna within two days after whiplash neck sprain injury Injury 1997 28 331 335 10.1016/S0020-1383(97)00027-2 9764227

[B32] Kongsted A Sorensen JS Andersen H Keseler B Jensen TS Bendix T Are early MRI findings correlated with long-lasting symptoms following whiplash injury? A prospective trial with 1-year follow-up Eur Spine J 2008 17 996 1005 10.1007/s00586-008-0687-9 18512085 PMC2518762

[B33] Pettersson K Hildingsson C Toolanen G Fagerlund M Bjornebrink J Disc pathology after whiplash injury. A prospective magnetic resonance imaging and clinical investigation Spine (Phila Pa 1976) 1997 22 283 287 9051890 10.1097/00007632-199702010-00010

[B34] Ronnen HR de Korte PJ Brink PR van der Bijl HJ Tonino AJ Franke CL Acute whiplash injury: is there a role for MR imaging?--a prospective study of 100 patients Radiology 1996 201 93 96 8816527 10.1148/radiology.201.1.8816527

[B35] Voyvodic F Dolinis J Moore VM Ryan GA Slavotinek JP Whyte AM Hoile RD Taylor GW MRI of car occupants with whiplash injury Neuroradiology 1997 39 35 40 10.1007/s002340050363 9121646

[B36] Karlsborg M Smed A Jespersen H Stephensen S Cortsen M Jennum P Herning M Korfitsen E Werdelin L A prospective study of 39 patients with whiplash injury Acta Neurol Scand 1997 95 65 72 10.1111/j.1600-0404.1997.tb00071.x 9059723

[B37] Spitzer WO Skovron ML Salmi LR Cassidy JD Duranceau J Suissa S Zeiss E Scientific monograph of the Quebec Task Force on Whiplash-Associated Disorders: redefining "whiplash" and its management Spine (Phila Pa 1976) 1995 20 Suppl 8 1 73 7604354

[B38] Vetti N Kråkenes J Damsgaard E Rørvik J Gilhus NE Espeland A MRI of the alar and transverse ligaments in acute whiplash-associated disorders 1-2 - a cross-sectional controlled study Spine (Phila Pa 1976) in press 10.1097/BRS.0b013e3181da21a9 21178847

[B39] Fejer R Jordan A Hartvigsen J Categorising the severity of neck pain: establishment of cut-points for use in clinical and epidemiological research Pain 2005 119 176 182 10.1016/j.pain.2005.09.033 16298059

[B40] Jensen MP Karoly P O'Riordan EF Bland F Jr Burns RS The subjective experience of acute pain. An assessment of the utility of 10 indices Clin J Pain 1989 5 153 159 10.1097/00002508-198906000-00005 2520397

[B41] Ohlund C Eek C Palmbald S Areskoug B Nachemson A Quantified pain drawing in subacute low back pain. Validation in a nonselected outpatient industrial sample Spine (Phila Pa 1976) 1996 21 1021 1030 8724085 10.1097/00007632-199605010-00005

[B42] Horowitz M Wilner N Alvarez W Impact of Event Scale: a measure of subjective stress Psychosom Med 1979 41 209 218 472086 10.1097/00006842-197905000-00004

[B43] Sterling M Jull G Kenardy J Physical and psychological factors maintain long-term predictive capacity post-whiplash injury Pain 2006 122 102 108 10.1016/j.pain.2006.01.014 16527397

[B44] Krakenes J Kaale BR Rorvik J Gilhus NE MRI assessment of normal ligamentous structures in the craniovertebral junction Neuroradiology 2001 43 1089 1097 10.1007/s002340100648 11792052

[B45] Krakenes J Kaale BR Moen G Nordli H Gilhus NE Rorvik J MRI assessment of the alar ligaments in the late stage of whiplash injury--a study of structural abnormalities and observer agreement Neuroradiology 2002 44 617 624 10.1007/s00234-002-0799-6 12136365

[B46] Ackelman BH Lindgren U Validity and reliability of a modified version of the neck disability index J Rehabil Med 2002 34 284 287 10.1080/165019702760390383 12440803

[B47] Pietrobon R Coeytaux RR Carey TS Richardson WJ Devellis RF Standard scales for measurement of functional outcome for cervical pain or dysfunction: a systematic review Spine (Phila Pa 1976) 2002 27 515 522 11880837 10.1097/00007632-200203010-00012

[B48] Vernon H Mior S The Neck Disability Index: a study of reliability and validity J Manipulative Physiol Ther 1991 14 409 415 1834753

[B49] Gabel CP Burkett B Neller A Yelland M Can long-term impairment in general practitioner whiplash patients be predicted using screening and patient-reported outcomes? Int J Rehabil Res 2008 31 79 80 10.1097/MRR.0b013e3282f44e10 18277208

[B50] Sterling M Kenardy J Jull G Vicenzino B The development of psychological changes following whiplash injury Pain 2003 106 481 489 10.1016/j.pain.2003.09.013 14659532

[B51] Modic MT Obuchowski NA Ross JS Brant-Zawadzki MN Grooff PN Mazanec DJ Benzel EC Acute low back pain and radiculopathy: MR imaging findings and their prognostic role and effect on outcome Radiology 2005 237 597 604 10.1148/radiol.2372041509 16244269

[B52] Kivioja J Jensen I Lindgren U Early coping strategies do not influence the prognosis after whiplash injuries Injury 2005 36 935 940 10.1016/j.injury.2004.09.038 16005003

[B53] Sterner Y Toolanen G Gerdle B Hildingsson C The incidence of whiplash trauma and the effects of different factors on recovery J Spinal Disord Tech 2003 16 195 199 12679676 10.1097/00024720-200304000-00013

[B54] Hartling L Brison RJ Ardern C Pickett W Prognostic value of the Quebec Classification of Whiplash-Associated Disorders Spine (Phila Pa 1976) 2001 26 36 41 11148643 10.1097/00007632-200101010-00008

[B55] Kivioja J Jensen I Lindgren U Neither the WAD-classification nor the Quebec Task Force follow-up regimen seems to be important for the outcome after a whiplash injury. A prospective study on 186 consecutive patients Eur Spine J 2008 17 930 935 10.1007/s00586-008-0675-0 18427841 PMC2443268

[B56] Carroll LJ Cassidy JD Cote P The role of pain coping strategies in prognosis after whiplash injury: passive coping predicts slowed recovery Pain 2006 124 18 26 10.1016/j.pain.2006.03.012 16644133

